# Transcriptional memory dampens heat shock responses in yeast: functional role of Mip6 and its interaction with Rpd3

**DOI:** 10.1093/g3journal/jkaf144

**Published:** 2025-06-19

**Authors:** Joan Serrano-Quílez, Ana Tejada-Colón, Carme Nuño-Cabanes, Susana Rodríguez-Navarro

**Affiliations:** Gene Expression and RNA Metabolism Laboratory, Instituto de Biomedicina de Valencia (CSIC), Jaume Roig, 11, Valencia 46010, Spain; Gene Expression and RNA Metabolism Laboratory, Instituto de Biomedicina de Valencia (CSIC), Jaume Roig, 11, Valencia 46010, Spain; Gene Expression and RNA Metabolism Laboratory, Instituto de Biomedicina de Valencia (CSIC), Jaume Roig, 11, Valencia 46010, Spain; Gene Expression and RNA Metabolism Laboratory, Instituto de Biomedicina de Valencia (CSIC), Jaume Roig, 11, Valencia 46010, Spain

**Keywords:** transcriptional memory, heat shock, Mip6, Rpd3, gene expression regulation, stress adaptation, proteostasis, yeast (*Saccharomyces cerevisiae*), chromatin regulation, mRNA metabolism

## Abstract

Cells must rapidly adapt to environmental fluctuations, including heat stress, to maintain homeostasis and ensure survival. A key adaptive mechanism is transcriptional memory, which enables cells to “remember” prior stress exposure and mount a faster or more controlled transcriptional response upon reexposure. However, the molecular mechanisms underlying transcriptional memory in the heat shock response (HSR) remain incompletely understood. Here, we investigate the role of the RNA-binding protein (RBP) Mip6 in regulating transcriptional memory during heat stress in *Saccharomyces cerevisiae*. Using qRT-PCR and RNA-seq, we demonstrate that prior heat shock exposure dampens the activation of heat-responsive genes upon a second stress, a phenomenon more pronounced in *mip6Δ* mutants. Our transcriptomic analyses reveal that transcriptional memory predominantly suppresses excessive gene expression changes, fine-tuning stress responses. Moreover, we identify a functional and physical interaction between Mip6 and the histone deacetylase Rpd3, a key regulator of transcriptional memory. Loss of both Mip6 and Rpd3 results in synthetic growth defects under heat stress and misregulation of Msn2/4-dependent transcripts, implicating Mip6 as a novel player in the coordination of chromatin and RNA-binding mechanisms during transcriptional memory. Additionally, we show that transcriptional memory modulates metabolic homeostasis and proteostasis. Collectively, our findings implicate Mip6 in the coordination of transcriptional memory in the HSR and reveal a novel link between the RBP Mip6 and the chromatin modifier Rpd3 HDAC in stress adaptation. These insights provide a foundation for further exploration of transcriptional memory mechanisms across diverse stress conditions.

## Introduction

Cells can sense environmental fluctuations and adapt to adverse conditions such as elevated temperatures. In response to stress, they activate a coordinated set of mechanisms aimed at minimizing and reversing the damage. These include transcriptional reprogramming, global inhibition of translation and growth, and metabolic adjustment (Jain and Roy 2009). One of the most studied mechanisms to respond to high temperature in yeast is the heat shock response (HSR), a specific transcriptional program that results in the overexpression of genes encoding stress-protective proteins, such as chaperones, and the silencing of genes related to cell growth (Morano *et al.* 2012; Santiago *et al.* 2020; Dea and Pincus 2024). The HSR is mainly regulated by the transcription factors Msn2/4 and Hsf1, which bind to regulatory elements of their target genes (Sorger and Pelham 1987; Martínez-Pastor *et al.* 1996; Zheng *et al.* 2016; Masser *et al.* 2019).

The transcriptional reprogramming triggered by stress can be memorized, enabling cells to respond faster to future stimuli, a phenomenon known as “transcriptional memory.” This phenomenon refers to the capacity of certain genes to retain transcriptional competence or repression after the original stimulus has subsided, allowing for a faster and more robust transcriptional response upon reexposure to similar stress. Transcriptional memory involves multiple mechanisms, including chromatin modifications, histone methylation and acetylation, promoter–enhancer interactions, and the action of RBPs that influence mRNA stability and decay (Fabrizio *et al.* 2019; Pascual-Ahuir *et al.* 2020; Sump and Brickner 2022; Li *et al.* 2023). Nuclear pores are also key elements in the establishment of transcriptional memory, serving as an anchoring structure for activators and repressors regulating memory (Light and Brickner 2013). In addition, nuclear pores facilitate the formation of gene loops, a phenomenon associated with transcriptional memory that consists of the interaction between the promoter and the 3′ end region of the genes (Tan-Wong *et al.* 2009; Hampsey *et al.* 2011). The integration of these mechanisms ensures that memory genes are rapidly activated or silenced based on prior exposure, optimizing the cell's adaptive response.

In yeast, the study of transcriptional memory has focused on the response to 2 different stimuli: the lack of inositol in the medium, which provokes transcriptional activation of the gene *INO1*, and the shift of the carbon source in the medium from glucose to galactose, which results in the expression of the galactose metabolism genes (Brickner *et al.* 2007; Light *et al.* 2013; D’Urso *et al.* 2016; Randise-Hinchliff *et al.* 2016; Lee *et al.* 2018). In these studies, it was described the role of the histone modifying complexes Rpd3L and Set1/COMPASS in the establishment of transcriptional repression memory (TREM), a process that regulates the rapid inactivation of silenced genes upon stress. At the beginning of transcription, RNAPolII recruits the Set1/COMPASS complex, which methylates H3K4. Then, the Pho23 subunit of the Rpd3L complex binds H3K4met, triggering histone deacetylation by Rpd3L and the repression of the genes. In the absence of the Pho23 subunit, the cells do not present TREM, which suggest this mechanism is key for maintaining the transcriptional memory (Lee *et al.* 2018).

We have recently demonstrated a functional role for the RBP Mip6 in the yeast HSR (Martín-Expósito *et al.* 2019). Under basal conditions, Mip6 localizes uniformly in the nucleus and cytoplasm, whereas upon heat shock, it accumulates in stress granules and p-bodies, while changing the binding to specific mRNA targets (Martín-Expósito *et al.* 2019). Mip6 preferentially binds Msn2/4-dependent mRNAs under optimal conditions, whereas under HS, it binds preferentially to nonstress-induced transcripts, such as genes encoding for ribosomal proteins (Martín-Expósito *et al.* 2019). In cells lacking Mip6, several Msn2/4-dependent transcripts, including genes related to trehalose metabolism, accumulate and are defectively exported to the cytoplasm, especially under HS, showing the dependence on Mip6 of these processes that are key to regulating the stress response (Martín-Expósito *et al.* 2019; Nuño-Cabanes *et al.* 2020). Trehalose is a disaccharide that serves a dual function in yeast by acting as a chemical chaperone and as an energy reserve that can be utilized during periods of cellular stress (Jain and Roy 2009).

Interestingly, we also found that Mip6 physically and genetically interacts with key regulators of mRNA synthesis and degradation, including the RNA polymerase II subunit Rpb1, the mRNA decay factors Rrp6 and Xrn1, and the general mRNA export factor Mex67. These interactions suggest a role for Mip6 in coordinating mRNA regulation under both stress and nonstress conditions. Although the *mip6Δ* single mutant does not exhibit a significant growth defect under stress, the observed imbalance in RNA homeostasis and export supports a role for Mip6 in fine-tuning the cellular response to thermal stress. Nonetheless, the precise mechanisms by which Mip6 selectively regulates its targets in response to environmental cues remain to be elucidated (Zander *et al.* 2016; Wang *et al.* 2020; García-Martínez *et al.* 2021).

The *MIP6* gene has a paralog, *PES4*, which also encodes a four–RNA-binding domain (RBD) protein with partially overlapping but distinct functional roles. Mip6 and Pes4 have been reported to act both redundantly and specifically to ensure proper expression and function of essential mRNAs during meiosis and sporulation in yeast, regulating their stability, translation, and subcellular localization. However, the *pes4Δ* mutant does not display the stress-related phenotypes observed in the *mip6Δ* mutant, suggesting functional divergence between the two proteins. Mip6 appears to play a unique role in fine-tuning transcriptional responses under environmental stress. Notably, only Mip6 has been shown to interact with Mex67, a key factor in the export of specific heat shock transcripts, further supporting the functional specialization of these yeast paralogs (Jin *et al.* 2017; Martín-Expósito *et al.* 2019).

Using transcriptomic, genetic, and biochemical approaches, we investigated the cellular changes that occur in *Saccharomyces cerevisiae* during the HSR following either a single heat shock (HS) or 2 sequential HS events. Our goal was to assess the existence and functional relevance of transcriptional memory by comparing cells subjected to a second stress after a 60-min recovery at 30°C (“memory” condition) with cells reexposed without recovery (“no-memory” condition). Previous studies have shown that key transcriptional adaptations occur within this timeframe, for example, genes that are strongly repressed following heat shock display transient downregulation, with expression levels returning to baseline approximately 60 min after treatment (Lu *et al.* 2009). This supports the use of a 60-min recovery period to probe transcriptional memory mechanisms. Furthermore, given Mip6's established physical and functional interactions with components of the transcriptional machinery, we examined whether Mip6 contributes to the regulation of transcriptional memory during heat shock (Lu *et al.* 2009).

## Materials and methods

### Microbiological and DNA recombinant techniques

*S. cerevisiae* strains and plasmids used in this work are listed in Tables 1 and 2, respectively. For yeast growth and gene deletions and transformation, standard methods were used (Martín-Expósito *et al.* 2019). The created strains were checked by Western blotting and/or PCR analysis. Yeast cultures were grown in liquid media at the indicated temperatures. The media used were yeast peptone dextrose (YPD) or synthetic complete (SC). For the growth assays, cells were diluted to a OD_600_ 0.1 and 260 µL of the cells was transferred to a 96-well plate, and the plated was inserted into a TECAN Spark Multimode Microplate Reader, where the cells were incubated at 30°C, 37°C, or 42°C until they reached stationary phase and OD_600_ of the samples were measured every 20 min.

**Table 1.** Yeast strains used during this study, with their background, their genetic description, and their source.

Strain	Genotype	Source
WT	MATa *his3*Δ*1 leu2*Δ*0 met15*Δ*0 ura3*Δ*0*	(Baker Brachmann *et al.* 1998)
*mip6Δ*	MATa *his3*Δ*1 leu2*Δ*0 met15*Δ*0 ura3*Δ*0 mip6::KANMX4*	EUROSCARF
*rpd3Δ*	MATa *his3*Δ*1 leu2*Δ*0 met15*Δ*0 ura3*Δ*0 rpd3::KANMX4*	EUROSCARF
*rpd3Δmip6Δ*	MATa *his3*Δ*1 leu2*Δ*0 met15*Δ*0 ura3*Δ*0 rpd3::LEU2 mip6::KANMX4*	This work
Rpd3-TAP	MATa *his3*Δ*1 leu2*Δ*0 met15*Δ*0 ura3*Δ*0 RPD3-TAP-HIS3*	Open BioSystems
Rpd3-TAP *mip6Δ*	MATa *his3*Δ*1 leu2*Δ*0 met15*Δ*0 ura3*Δ*0 RPD3-TAP-HIS3 mip6::LEU2*	This work

**Table 2.** Yeast plasmids used during this study, with their description and their source.

Plasmid	Description	Source
p*ADH1pr*-GFP	Yeast plasmid for protein expression regulated by *ADH1* promoter and tagged in the C-terminal with a GFP epitope (*URA3* marker)	(Taberner *et al.* 2009)
p*ADH1pr*-Mip6-GFP	Yeast plasmid for Mip6 expression regulated by *ADH1* promoter and tagged in the C-terminal with a GFP epitope (*URA3* marker)	(Martín-Expósito *et al.* 2019)
p*ADH1pr*-Mip6W_442_A-GFP	Yeast plasmid for Mip6W_442_A expression regulated by *ADH1* promoter and tagged in the C-terminal with a GFP epitope (*URA3* marker)	(Martín-Expósito *et al.* 2019)

### Stress treatments

For heat shock treatments, cells were grown at optimal conditions until exponential phase and were split into two different flasks: one for the heat shock treatment and the other one for the nonstress control. In the heat shock treatment flask, one part of the volume of the corresponding medium at 51°C was added so that the resulting temperature of the mixture was 39°C. Immediately, the cells were incubated at 39°C for 20 min. In the nonstress control flask, one part of the volume of the corresponding medium at 30°C was added.

For the oxidative stress treatment, a yeast cell survival protocol was followed (Tran and Green 2019). Yeast cells were grown in YPD until exponential phase equivalent. In the oxidative stress treatment tube, 4 mM H_2_O_2_ was added, and both tubes were incubated at 30°C for 30 min. Immediately after the incubation, cells were centrifuged and resuspended in YPD.

### Protein immunoprecipitation and Western blotting

Protein immunoprecipitation was performed using GFT-TRAP (ChromoTek), following the guidelines the company provides with certain modifications. Cells grown until exponential phase were resuspended in lysis buffer (10 mM Tris–HCl pH 7.5, 150 mM NaCl, 0.5 mM EDTA, 0.5× cOmplete (Roche), 0.5% (v/v) NP-40). Cells were broken by mixing with 0.5 mm acid-washed glass beads. The cell lysate was obtained by centrifugation at 13,000 rpm for 10 min at 4°C and incubated for 1 h at 4°C with GFT-TRAP beads previously equilibrated. Proteins bound to the beads were eluted with LB 4× by incubation at 95°C for 10 min. For Western blotting, the antibodies used were α-GFP (Roche) and α-TAP (Invitrogen).

### Protein extraction

To measure the levels of Hsp12, a trichloroacetic acid (TCA) precipitation protocol was used for protein extraction. Yeast cultures (2–15 mL) were grown to OD_600_ 0.3–0.6 and centrifuged (3,000 rpm, 4°C, 5 min). Pellets were resuspended in 1 mL 20% (v/v) TCA, transferred to 2 mL tubes, and centrifuged (13,000 rpm, 4°C, 1 min). Pellets were washed with 1 mL 1 M Tris (no pH adjustment) and centrifuged again. Cells were resuspended in 2× loading buffer (100 mM Tris–HCl, pH 6.8, 3.68% SDS, 16% glycerol, 0.08% bromophenol blue, 200 mM DTT) and boiled at 95°C for 2 min. After adding 100 μL acid-washed glass beads, samples were lysed using a Precellys Evolution homogenizer (6,500 rpm, 30 s, 2 cycles, 15 s pause) and centrifuged (4,000 rpm, RT, 5 min). Supernatants were stored at −20°C and boiled at 95°C for 5 min before use. For Western blotting, the antibodies used were α-Pgk1 (Invitrogen) and α-Hsp12 (from Dr J. Buchner).

### Trehalose extraction and quantification

Yeast cells (10 mg) were centrifuged, washed, and stored at −80°C. Pellets were resuspended in 0.25 mL Na₂CO₃ (250 mM) and incubated at 95°C for 4 h. After adding acetic acid (0.15 mL, 1 M) and sodium acetate (0.6 mL, 0.2 M, pH 5.2), samples were centrifuged (12,000 rpm, 1 min), and supernatants were collected.

Samples (150 µL) were split: one incubated with trehalase (8.4 mU, 5 µL) and one as a blank. Reactions were incubated overnight at 37°C, boiled (5 min), and centrifuged (30 s, max speed).

A glucose standard curve (0–10 µg) was prepared (100 µL final volume). The reaction mixture contained 80 mM potassium phosphate buffer (pH 7), 65 µg/mL glucose oxidase, 5 µg/mL peroxidase, and 300 µg/mL o-dianisidine (added last). Samples (100 µL) were mixed with 400 µL of the reaction mixture, incubated at 30°C (15 min), and stopped with 500 µL HCl (6 M), and absorbance was measured at 540 nm using a Varioskan LUX. Intracellular trehalose was expressed as µg trehalose/mg cells.

### Gene expression analysis by qPCR

Total RNA was isolated by hot acid phenol extraction (Collart and Oliviero 2001). Ten microgram of the total RNA was incubated with DNase I recombinant RNase free (Roche) and was then purified by phenol–chloroform extraction. cDNA was synthesized using the PrimeScript RT reagent Kit (Takara). Relative RNA levels were measured by quantitative PCR (qPCR) using the QuantStudio 5 Real-Time PCR System (Thermo Scientific) or the CFX Opus 96 Real-Time PCR System (Bio-Rad). Specific pairs of primers that were used in the analysis are listed in Table 3. TB Green Premix Ex Taq (Tli RNase H Plus; Takara) was used in a set volume of 10 µL per reaction. The relative quantity of each PCR amplicon was measured following the 2^−ΔΔCT^ method (Livak and Schmittgen 2001) using *SCR1* as a reference gene for normalization. Three technical replicates were performed for each sample.

**Table 3.** Primers employed during this study, with their sequence in the 5′ → 3′ direction.

Primer	5′ → 3′ sequence
*HSP12* ORF FW	CTTCCAAGGTGTCCACGACT
*HSP12* ORF RV	ACATATTCGACGGCATCGTT
HSP78 ORF FW	TTGAACTCCATGGCAACTTTC
HSP78 ORF RV	CGCCTTCTTCATGATTTGGT
HSP26 ORF FW	GGTCAAGGTCAAAGGAGAGCA
HSP26 ORF RV	TGTCAAAACACCATTTGCGTA
ACC1 ORF FW	TAAACTAGAGGAGTCCCCGT
ACC1 ORF RV	TAATTTCTTTCACGGCGGCA
FAA1 ORF FW	GTTCCAGTTGGGAAAGCCGC
FAA1 ORF RV	CCCTCCAACCCATAGCATTT
TPS1 ORF FW	GGCGCAACTGACCTCGTCTT
TPS1 ORF RV	CTTCCAACGCCGTGACCAGC
TPS2 ORF FW	TTGTGTCACGCAGCTGCCCT
TPS2 ORF RV	CAACGTGTTGCTCGTACTCG
*CTT1* ORF FW	GAACCATGACTGGGTCTTCA
*CTT1* ORF RV	GAAGGAATGACCAGAGTACG
*SCR1* FW	GAGTTTTATCCAGCGTCAGC
*SCR1* RV	GGTTCAGGACACACTCCATC

### Processing of mRNA datasets

For RNA-seq analyses, total RNA isolated by hot acid phenol extraction, as above, was submitted to Macrogen, Inc to be processed, using the Illumina TruSeq protocol. Between 40 and 60 million reads were obtained from each sample. The quality of the raw sequencing data was checked by FastQC and trimmomatic program was used to withdraw the adapter sequences and the bases with a low base quality from the ends. Moreover, the bases of reads that had a window size lower than 4 and a mean quality lower than 15 and the reads shorter than 36 bp were also trimmed. After trimming, the quality of the samples was checked again by FastQC. Trimmed reads were mapped using sacCer3 as a reference genome with HISAT2, splice-aware aligner, and StringTie. The abundance of each gene and transcript was calculated in the read count for each sample.

The NOISeq R package (Tarazona *et al.* 2015) was used to perform the quality control of count data. Counts were normalized via TMM (Bullard *et al.* 2010), and a low count filtering was applied with the NOISeq *cpm* method (with cpm = 1). Hidden batch effects were removed by ARSyN (Nueda *et al.* 2012). In total, we obtained gene expression values for 6,172 genes. A principal component analysis (PCA) was applied to these normalized counts to get a first glimpse over the transcriptomic changes.

### Differentially expressed genes analysis

The NOISeq method (Tarazona *et al.* 2015) was used on the normalized counts matrix to identify differentially expressed genes in 1 to 1 comparisons. *mip6*Δ and wild-type (WT) strains at 15 and 20 min were always compared to the initial reference of the same strain. These pairwise fold changes were then represented using Sankey diagrams. Fold changes obtained by NOISeq were organized into different categories. Genes with absolute fold changes greater than 1.2 were classified as slightly differentially expressed (“up” or “down”), while those with absolute fold changes over 2 were considered strongly differentially expressed (“strong up” or “strong down”). This categorization resulted in 5 groups. Genes showing no significant changes were labeled as “unchanged.”

### Study of gene expression patterns

Gene expression dynamics were analyzed using maSigPro (Conesa *et al.* 2006), applying a regression model that incorporated both linear and quadratic terms for time, as well as their interactions with memory and strain. For each *i* gene, its expression $Y_{i}$ is determined by Eq. 1.


(1) $$\begin{array}{rl} Y_{i} & =\beta_{0}+\beta_{\text{M}emory}+\beta_{\text{S}train}+\beta_{\text{M}emory.Strain}+\beta_{\text{T}ime}\cdot t+\beta_{\text{T}ime.Memory}\cdot t\\ & \;+\beta_{\text{T}ime.Strain}\cdot t+\beta_{\text{T}ime.Memory.Strain}\cdot t+\beta_{\text{T}ime^{2}}\cdot t^{2}+\beta_{\text{T}ime^{2}.Memory}\cdot t^{2}\\ & \;+\beta_{\text{T}ime^{2}.Strain}\cdot t^{2}+\beta_{\text{T}ime^{2}.Memory.Strain}\cdot t^{2}+\varepsilon_{i} \end{array}$$

A backward stepwise regression approach was used to refine the model, progressively removing nonsignificant terms while maintaining interactions essential for biological interpretation. Genes with no significant association with time were eliminated based on both their linear and quadratic terms ($\beta_{Time}$ and $\beta_{Time^{2}}$ > 0), thus doing all further analysis with 3,929 genes.

### Filtering and classification of genes

Despite the initial filtering step that removed genes lacking time-dependent expression patterns, the classification strategy did not consider the quadratic interaction terms to simplify the number of possible expression profiles, since there was a high correlation between both terms. However, the linear model was not redefined after excluding these terms, ensuring that the structure of the statistical framework remained consistent with the initial full model.

### Categorization of gene expression patterns

Each coefficient in the model represents a specific effect on gene expression. The term $\beta_{Time}\;$ captures the linear trend of gene expression over time, with positive values indicating induction and negative values indicating repression. The inclusion of a quadratic term $\beta_{Time^{2}}$ allows for nonlinear trends in expression changes. The coefficients $\beta_{Memory}$ and $\beta_{Strain}\;$ describe the baseline effects of memory and strain, respectively, which indicate whether a gene is expressed at different levels depending on these conditions.

Interactions between factors were incorporated to assess how gene expression dynamics were modulated by memory and strain. The term $\beta_{Time.Memory}$ represents the effect of memory over time, meaning that if the absolute value of $\beta_{Time}+\beta_{Time.Memory}\;$ is greater than $\beta_{Time}$ alone, the memory effect enhances the response. Conversely, if $|\beta_{Time}+\beta_{Time.Memory}\left|<\right|\beta_{Time}|$ , the memory effect dampens the response. If the sign of $\beta_{Time}+\beta_{Time.Memory}\;$ is opposite to that of $\beta_{Time}$ , the effect is classified as a reversal of the trend.

A similar rationale applies to the strain effect, as determined by $\beta_{Time.Strain}$ . If $\beta_{Time.Strain}+\beta_{Time}\;$ increases the absolute value relative to $\beta_{Time}$ , the strain effect is classified as enhancement, whereas a reduction in magnitude is classified as dampening. A sign change is indicative of a complete reversal of the expression trend.

The interaction term $\beta_{Time.Memory.Strain}$ accounts for cases where memory and strain effects combine to produce alterations in time-dependent expression that cannot be explained by their independent effects alone. If $\beta_{Time.Memory.Strain}\;$ is significantly different from zero, this suggests a synergistic or antagonistic interaction between memory and strain. The classification into enhancement, dampening, or reversal in these cases depends on the net effect of all interacting coefficients. Specifically, when reinforces the combined influence of memory and strain, the gene is classified as further enhanced or dampened, depending on whether the net change increases or decreases absolute expression levels. In contrast, if it causes a shift that counteracts the expected trend from memory or strain effects alone, the gene is classified as reversed.

### Gene Ontology (GO) enrichment analysis

To investigate the functional implications of differentially expressed genes, a GO enrichment analysis was performed using the clusterProfiler package (Ashburner *et al.* 2000; Wu *et al.* 2021; The Gene Ontology Consortium *et al.* 2023). Genes from each category were tested for enrichment in biological process (BP) terms using *S. cerevisiae* annotations from org.Sc.sgd.db. The analysis was conducted using Fisher's exact test with Benjamini–Hochberg correction (FDR < 0.05). ORF names were validated against the database before analysis, ensuring accurate annotation. The results provided insights into the biological processes associated with memory, strain, and their interactions over time.

## Results

### A 60-min recovery prior to sequential heat exposure dampens the activation of heat shock genes

**Fig. 1.**
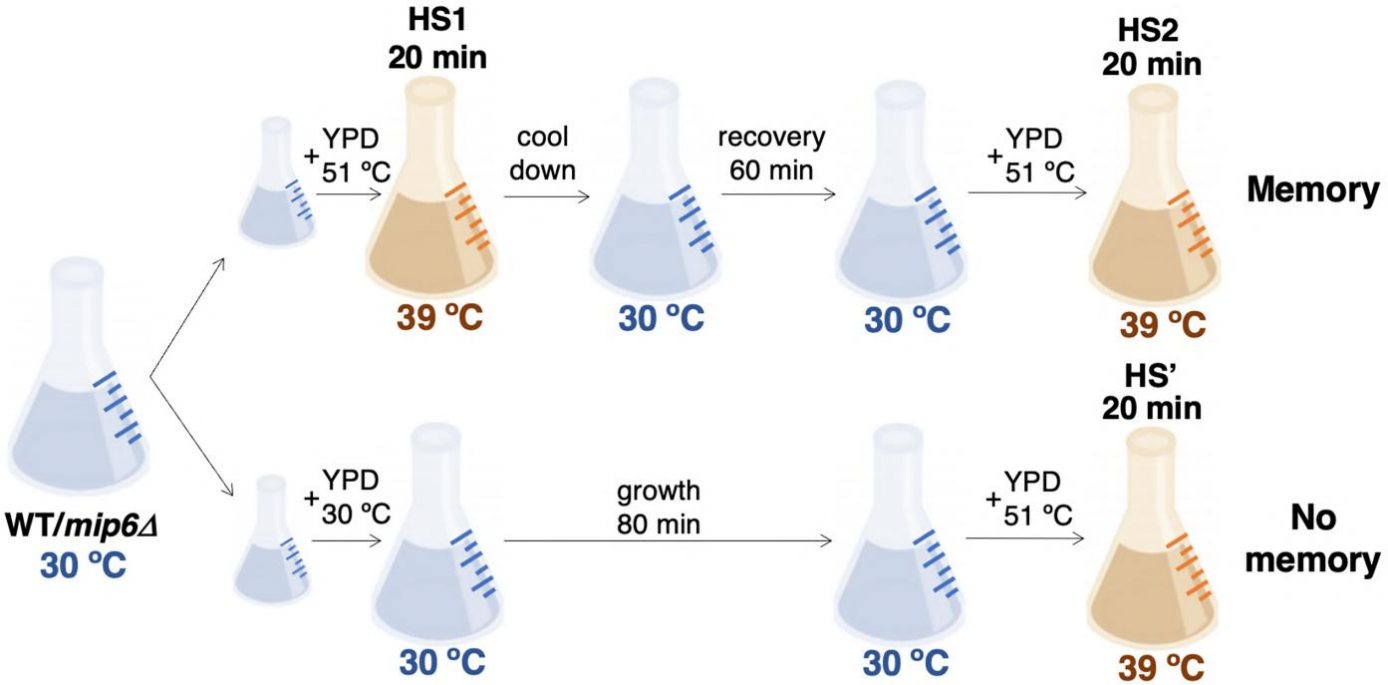
Experimental design for the study of the transcriptional memory associated with HS. A cell culture was split into two flasks. One of them (memory) was exposed to a first 20-min heat shock and, after a 60-min recovery phase at optimal conditions, to a second 20-min heat shock. In the meantime, the other flask (no-memory) was only exposed to a single 20-min heat shock. The heat shocks were performed by adding media at 51°C.

Despite growing interest in stress adaptation, the transcriptional memory induced by heat stress in yeast remains incompletely characterized. To address this, we conducted a heat shock memory experiment using an overnight culture grown at 30°C, which was split into two conditions: “no-memory” and “memory” (Fig. 1). The “memory” sample was mixed with 51°C-tempered medium to rapidly raise the temperature to 39°C, inducing the first heat shock (HS1) for 20 min. The culture was then returned to 30°C for a 60-min recovery, followed by a second 20-min heat shock at 39°C (HS2). In parallel, the “no-memory” culture was refreshed with 30°C medium and maintained for 80 min before undergoing a single 20-min heat shock at 39°C. Samples were collected at 0, 5, 10, 15, and 20 min during the final heat shock from both conditions for RT-qPCR analysis. Experiments were performed in triplicate for both WT and *mip6Δ* strains (Fig. 1).

We used a colony-forming unit (CFU) assay to assess whether heat shock, recovery, or transcriptional memory affected yeast cell survival. CFUs were measured at 0 and 20 min of heat shock, and values were normalized to the highest viability observed at each time point. No significant differences using two-tailed paired *t*-tests comparing memory and nonmemory conditions in viability were detected between WT and *mip6Δ* strains, or between memory and nonmemory conditions (Supplementary Fig. 1). These results indicate that although a mild viability reduction occurs in *mip6Δ* cells upon heat shock, the heat shock protocols used—either followed by a recovery period or not—do not appreciably impact the colony-forming ability of yeast cells.

**Fig. 2.**
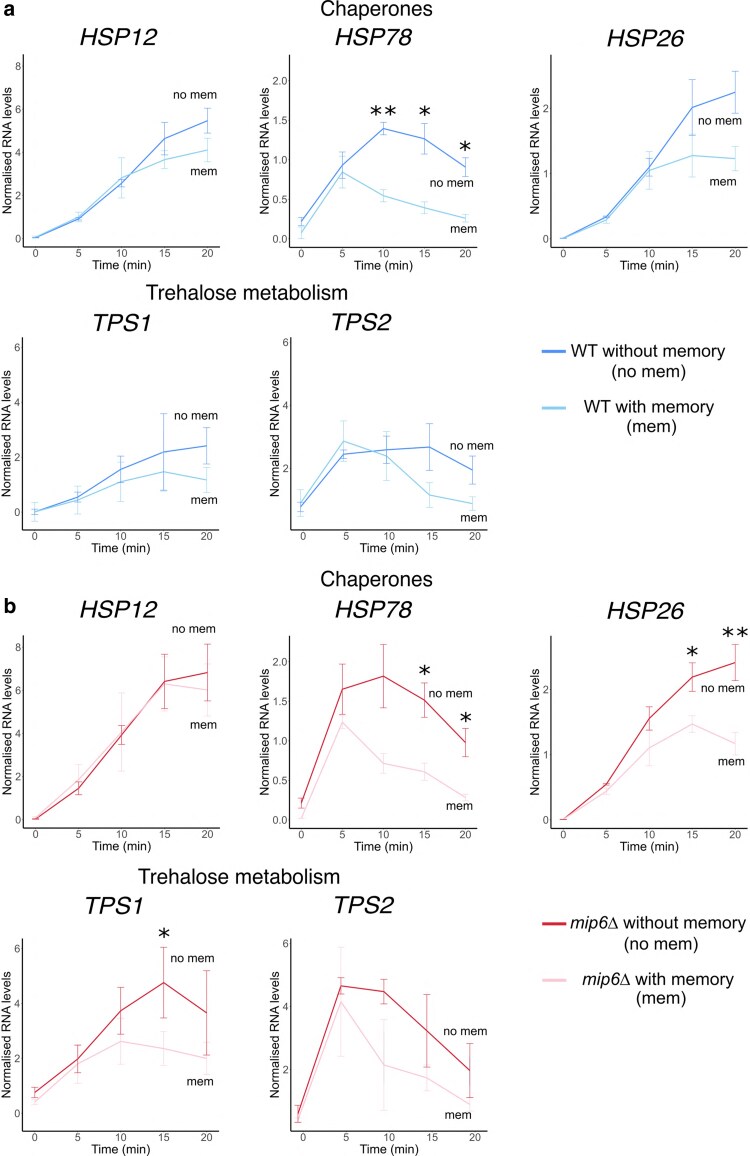
The “no-memory” condition resulted in a higher activation of heat-responsive genes. *HSP12*, *HSP78*, *HSP26*, *TPS1*, and *TPS2* RNA levels measured by qRT-PCR experiments from WT (a) and *mip6*Δ (b), using *SCR1* as a reference gene. Values were calculated by using the ΔΔCt method, using *SCR1* gene as a reference gene. Sample differences were analyzed using a two-tailed paired *t*-test in R. Data are presented as mean ± standard error (SE) of 3 biological replicates. Statistical significance is indicated as follows: *P* < 0.05 (*), *P* < 0.01 (**), and *P* < 0.001 (***).

We used RT-qPCR to analyze the activation of specific HSR genes, including *HSP12*, *HSP78*, *HSP26*, *TPS1*, and *TPS2*. In WT cells, gene activation was detected within 5–10 min of the HS2, regardless of prior heat exposure (Fig. 2a). However, at later time points (15–20 min), expression levels began to diverge depending on the gene. Notably, *HSP78* showed a statistically significant reduction in activation at 15–20 min (Fig. 2a). These results suggest that a 60-min recovery period at 30°C is not sufficient to dampen early transcriptional activation during a subsequent heat shock.

We then performed the same analysis in *mip6Δ* cells (red lines, Fig. 2b). As expected from previous results, loss of *MIP6* resulted in overall higher expression levels of heat shock genes compared to WT (Supplementary Fig. 2). The effect of transcriptional memory in *mip6Δ* cells resembled that observed in WT, showing a general trend of reduced gene activation at later time points (15–20 min) following the HS2 (Fig. 2b). However, in *mip6Δ*, a greater number of genes exhibited statistically significant reductions in activation during prolonged heat exposure. Specifically, while only *HSP78* showed significant dampening in WT cells, *TPS1* and *HSP26* also displayed significant reductions in *mip6Δ* cells (Fig. 2b). These findings suggest that Mip6 influences the extent and specificity of transcriptional memory effects during the HSR.

### Global analysis of transcriptional memory following mild heat shock

To extend our preliminary qPCR analysis to a genome-wide scale, we performed RNA-seq following the experimental design shown in Fig. 1. Samples were collected at 0, 15, and 20 min after the onset of heat stress, corresponding to time points that showed notable gene activation differences in the qPCR analysis.

**Fig. 3.**
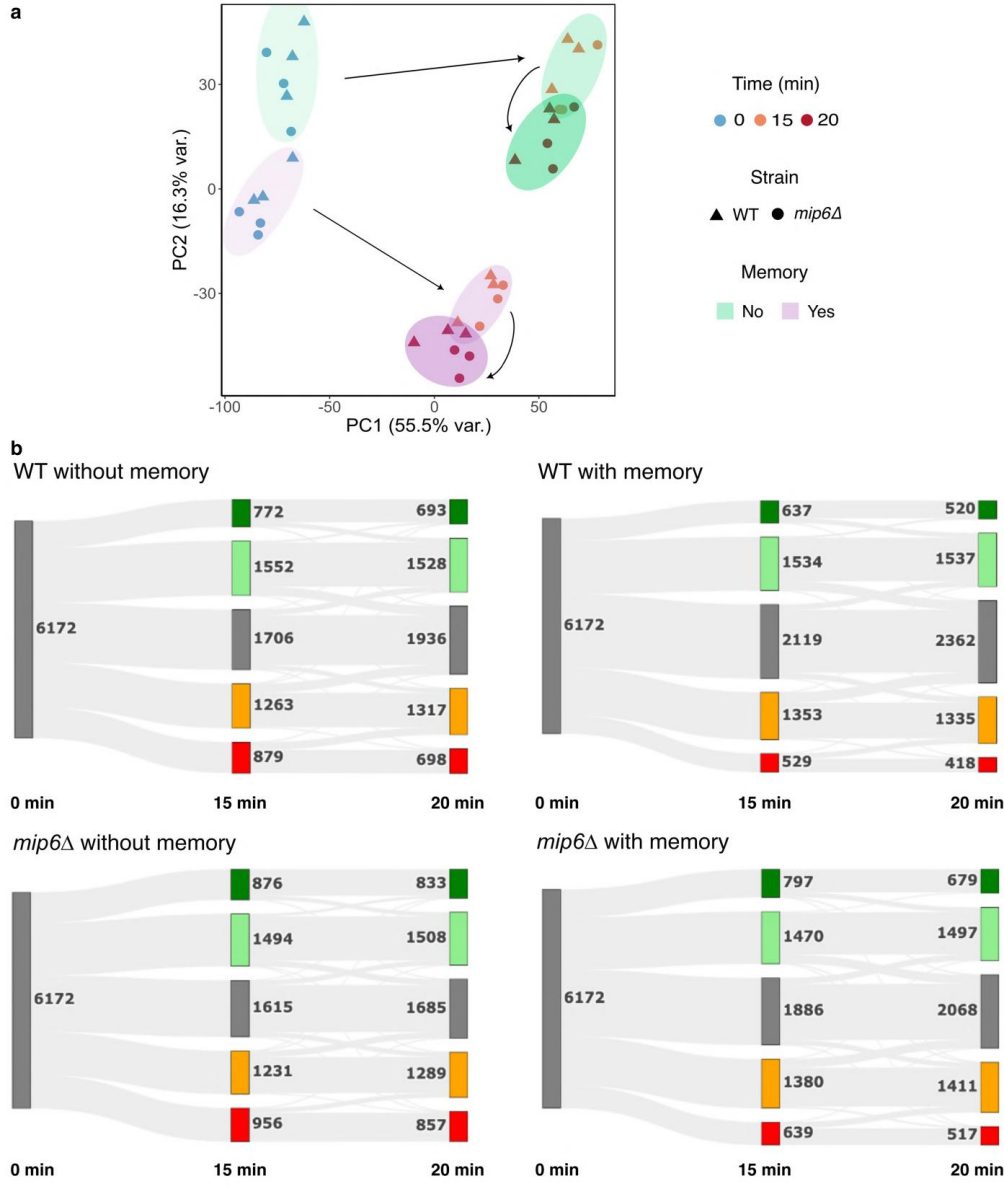
Heat shock stress response is different when yeast cells have already suffered a previous heat exposition. a) PCA score plot from the RNA-seq analysis of the WT and *mip6*Δ collected at different times during a heat shock treatment (0, 15, or 20 min at 39°C). The samples were previously exposed to a 20-min heat shock at 39°C with a 60-min recuperation period at 30°C (memory) or were previously grow only under optimal conditions (no-memory). Three biological replicates were carried out. b) Sankey plots representing changes in gene expression along time under heat stress. Genes are classified according to their expression level when compared to the initial time in the same strain and condition. In dark green, genes with a fold change higher than 2. In light green, genes displaying a fold change between 1.2 and 2. In gray, those genes with a fold change lower than 1.2 and higher than −1.2 or not significantly affected. In orange, genes with a fold change between −1.2 and −2. In red, those with a fold change lower than −2.

As an initial overview of the transcriptomic data, we performed PCA to identify the main sources of variance among samples (Fig. 3a). PCA revealed a clear separation of transcriptomes, with principal component 1 (PC1) accounting for 55.5% of the variance and primarily reflecting time and temperature. PC1 distinguished samples grown under optimal conditions (0 min, blue) from those exposed to heat stress (15 and 20 min, orange and red, respectively), indicating that environmental conditions were the dominant drivers of transcriptional variation.

Principal component 2 (PC2), which explained 16.3% of the variance, separated samples based on their prior heat exposure (“memory” vs “no-memory”), suggesting that transcriptional memory contributed substantially—over one-fifth—to the observed differences when cells were allowed to recover for 60 minutes. In contrast, strain identity and biological replicates contributed minimally to the variance, as nearly 75% of transcriptomic variation could be attributed to the combined effects of heat stress and transcriptional memory.

To evaluate the global impact of transcriptional memory on gene expression, we visualized transcriptomic changes using Sankey plots (Fig. 3b). Out of the 6,172 genes analyzed, 1,706 genes showed no induction or repression after 15 min of heat shock in the no-memory condition, whereas this number increased to 2,119 in the memory condition for WT cells. In *mip6Δ* cells, 1,615 genes showed no induction or repression in the no-memory condition, and 1,886 in the memory condition.

This buffering effect of transcriptional memory was also evident among strongly activated and repressed genes (depicted in dark green and red, respectively), which decreased from 772 to 637 genes in WT cells and from 876 to 797 in *mip6Δ* cells. A similar trend was observed when comparing 0 and 20 min of heat shock. Interestingly, *mip6Δ* consistently exhibited a higher number of strongly regulated genes at both time points and in both memory and no-memory conditions (Fig. 3b). These findings reinforce the role of Mip6 in fine-tuning mRNA levels and suggest that it contributes to the precise modulation of transcriptional responses to heat stress across a specific subset of genes

### Transcriptional memory predominantly dampens gene expression during heat shock

**Fig. 4.**
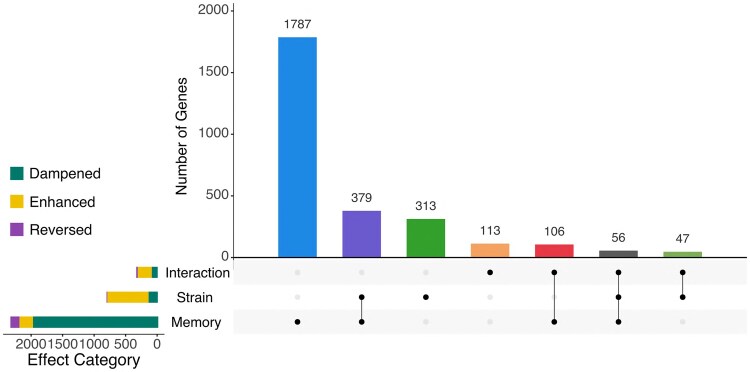
UpSet plot showing the overlap between gene groups that exhibit significant changes in expression due to memory, strain, or their interaction effects. The vertical bars represent the number of genes in each overlapping category. The horizontal bars categorize genes based on their response to experimental conditions, with colors representing different types of regulatory changes: enhancement, dampening, or reversal. Specifically, each horizontal bar corresponds to a different regression coefficient: (1) $\beta_{Time.Memory}$ (effect of memory on time-dependent expression), (2) $\beta_{Time.Strain}$ (effect of strain on time-dependent expression), and (3) $\beta_{Time.Memory.Strain}$ (interaction effect of memory and strain on time-dependent expression). Only genes that exhibit significant changes for each factor are included in the classification.

To study how transcriptional memory, strain background, or the interaction of both factors affected gene expression dynamics during heat shock, genes that showed significant temporal changes in expression $\beta_{Time}$ and $\beta_{Time^{2}}$ ≠ 0)—defined by nonzero coefficients for time ($\beta_{Time}$) and/or quadratic time ($\beta_{Time^{2}}$)—regardless of whether they were induced or repressed, were classified as (1) enhanced, genes whose expression trend over time was amplified by any of these factors; (2) dampened, genes whose trend persisted but at a lower intensity; and (3) reversed, genes that changed the direction of their temporal expression profile. The classification for each regression coefficient was based on comparisons of nested models: for memory, we compared the sum of coefficients ($\beta_{Time}$ + $\beta_{Time\cdot Memory}$ ) to $\beta_{Time}$ ; for strain, we compared ($\beta_{Time}$ + $\beta_{Time\cdot Strain}$ ) to $\beta_{Time}$ ; and for the interaction, we compared the full model—including $\beta_{Time\cdot Strain\cdot Memory}$ —with a reduced model that excluded this term (Supplementary File 1). Only genes with statistically significant coefficients for each factor were included. As shown in Fig. 4, transcriptional memory alone significantly affected 1,787 genes (vertical blue bar). A total of 798 genes were influenced by strain effects, with 313 genes affected solely by strain, and 379 affected by both strain and memory. An additional 113 genes were significantly affected only by the interaction between memory and strain. These results suggest that the presence of *MIP6* is required for a coordinated transcriptional response in a specific subset of genes under defined environmental conditions.

**Fig. 5.**
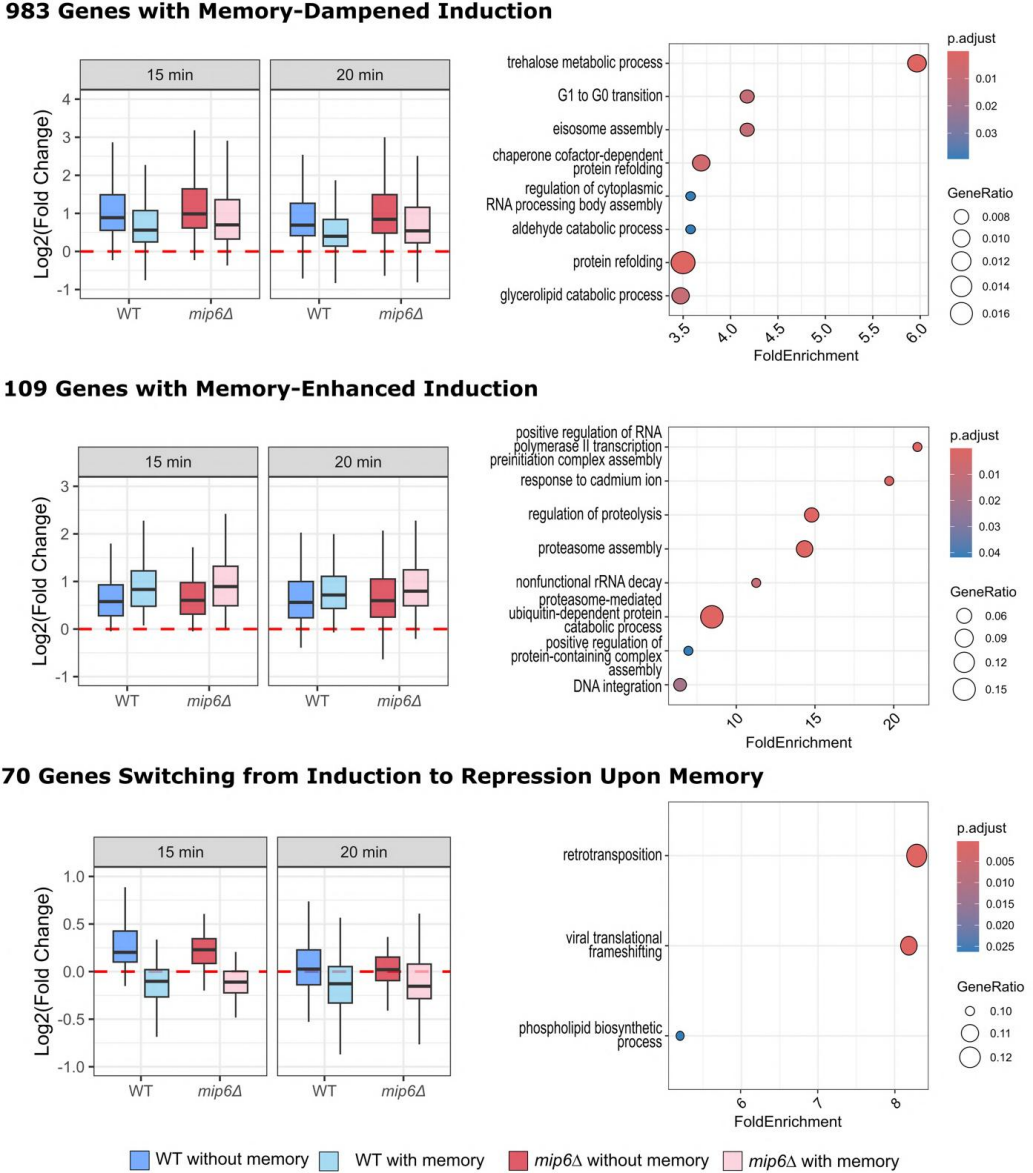
Expression patterns and functional enrichment of HS-induced genes classified by memory effect. HS-induced genes were grouped according to the effect of transcriptional memory into three categories: dampened, enhanced, or reverted. On the left, boxplots display the distribution of log₂ fold changes at 15 and 20 min after heat shock, computed using the NOISeq method, across 4 experimental conditions: WT with and without memory and *mip6Δ* with and without memory. Red dashed lines indicate the baseline (log₂FC = 0). Each panel corresponds to a distinct memory effect category. On the right, dot plots show the most significantly enriched GO-BP terms for each gene set. Enrichment was performed using the clusterProfiler::simplify function, and terms were ranked by fold enrichment. Only terms with adjusted *P*-values below 0.05 were retained. Full enrichment tables are provided in Supplementary File 2.

**Fig. 6.**
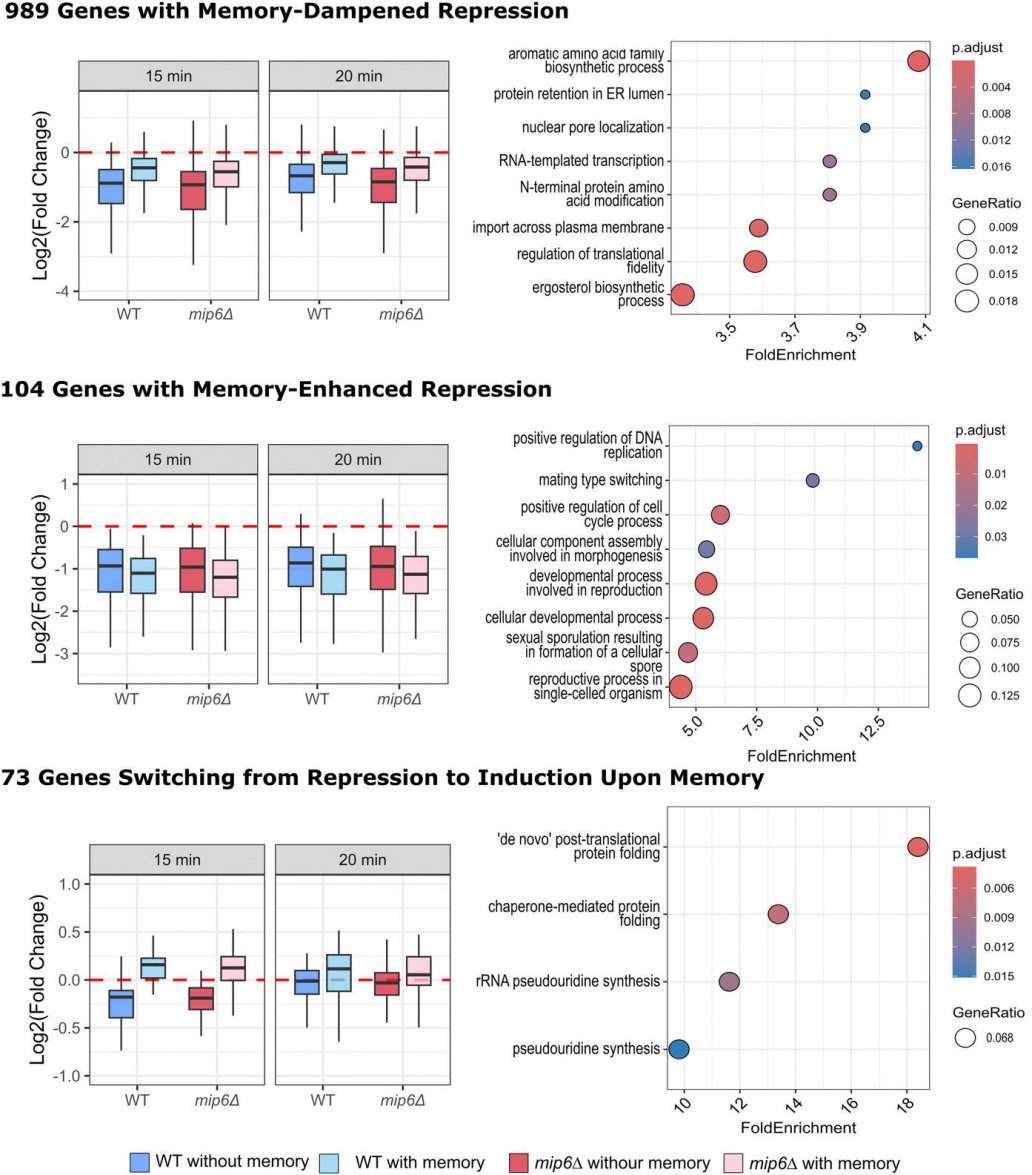
Expression patterns and functional enrichment of HS-repressed genes classified by memory effect. HS-repressed genes were classified into dampened, enhanced, or reverted categories based on the effect of transcriptional memory. Log₂ fold changes at 15- and 20-min postheat shock were computed using the NOISeq method and are shown as boxplots (left), across the same 4 conditions: WT and *mip6Δ*, with or without memory. Red dashed lines indicate the baseline (log₂FC = 0). Dot plots (right) display the most enriched GO-BP terms for each group, obtained using clusterProfiler::simplify and ranked by fold enrichment. Only terms with adjusted *P*-values below 0.05 were included. Complete enrichment results are provided in Supplementary File 2.

We further analyzed gene expression dynamics by focusing on time-responsive genes and the effect of transcriptional memory on their expression trajectories. Genes with significant temporal variation were grouped into two broad categories: heat shock-induced (HS-induced; Fig. 5), with increased expression over time, and heat shock-repressed (HS-repressed; Fig. 6), with decreased expression. Within each category, genes were further subdivided based on the effect of transcriptional memory over time, as captured by the interaction term $\beta_{Time\cdot Memory}$ .

Our analysis revealed that memory predominantly dampens gene expression changes in both HS-induced and HS-repressed genes. Specifically, 983 HS-induced and 989 HS-repressed genes exhibited dampened expression profiles in the presence of memory (Figs. 5 and 6, upper panels, left side). To elucidate the functional roles of these genes, we performed GO enrichment analysis [GO-Biological Process (GO-BP); Supplementary File 3] on each subset (Figs. 5 and 6, upper panels, right side). Among memory-dampened HS-induced genes, we observed enrichment in terms such as trehalose metabolic process, G1 to G0 transition, eisosome assembly, and protein refolding, suggesting a role in regulating stress adaptation and metabolic transitions. In contrast, memory-dampened HS-repressed genes were enriched in aromatic amino acid biosynthetic process, protein retention in the ER lumen, and nuclear pore localization, pointing to regulation of biosynthetic and trafficking pathways.

Memory-enhanced expression patterns were less frequent, involving 213 genes across both categories (Figs. 5 and 6, middle panels). Among these, HS-induced genes were enriched for proteolysis, proteasome activity, and mitochondrial organization, whereas HS-repressed genes were linked to meiosis, reproduction, and cell differentiation.

The least represented group was the memory-inverted category, comprising 143 genes (Figs. 5 and 6, lower panels). Despite their lower number, the enriched GO terms pointed to roles in retrotransposition (HS-induced) and chaperone-mediated protein folding (HS-repressed).

Together, these findings indicate that transcriptional memory predominantly acts by dampening gene expression changes over time, likely serving to prevent excessive induction or repression of stress-responsive genes. Moreover, the selective enhancement of pathways related to proteostasis—such as proteasome-mediated degradation, chaperone-assisted folding, and posttranslational protein processing—alongside the dampening of biosynthetic and cell cycle—associated processes, suggests that memory contributes to stress resilience by modulating protein quality control and conserving energy under recurrent stress conditions.

### Persistence of stress-induced proteins under memory conditions

**Fig. 7.**
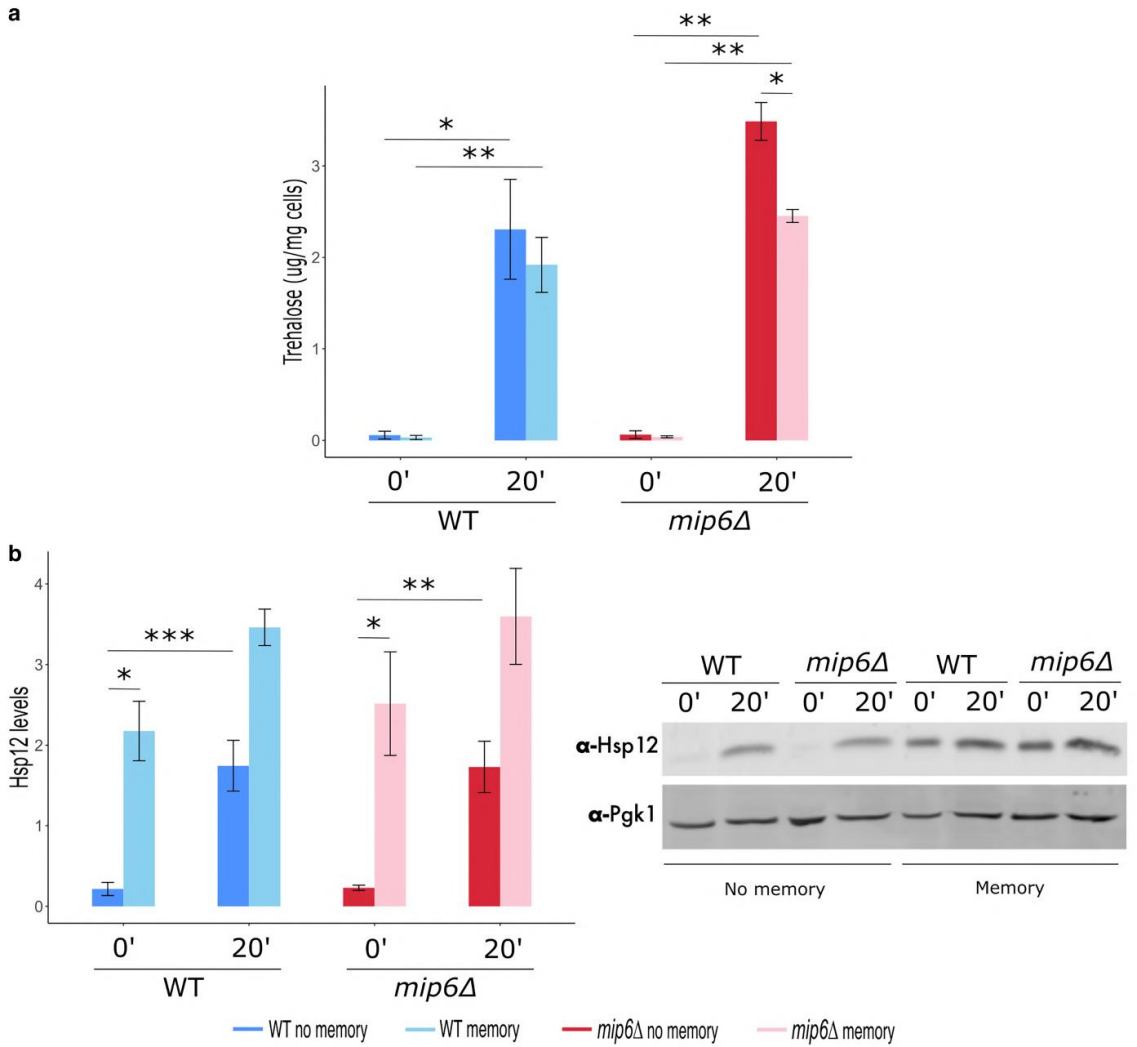
Effect of memory on different gene expression products. a) Trehalose levels were measured by enzymatic hydrolysis with trehalase. b) Hsp12 protein levels were quantified following TCA precipitation and Western blotting. Western blot images show Hsp12 and Pgk1 protein levels, with Pgk1 used as a loading control for normalization. Statistical differences between conditions were analyzed using a two-tailed paired *t*-test in R. Data represent the mean ± SE of 3 biological replicates. Statistical significance is denoted as follows: *P* < 0.05 (*), P < 0.01 (**), and *P* < 0.001 (***).

To further investigate the role of transcriptional memory in promoting metabolic and protein homeostasis, we measured intracellular trehalose levels. This analysis was motivated by the enrichment of the GO term GO:0005991 (trehalose metabolic process)—which includes *TPS2*, *NTH2*, *TPS1*, *CDC28*, *UGP1*, and *TSL1*—among HS-induced genes whose expression was dampened by memory (Figs. 5 and 6). As shown in Fig. 7a, trehalose accumulation increased after 20 min of heat shock but was attenuated in the memory condition. This dampening effect was particularly pronounced in the *mip6Δ* strain, where trehalose levels were significantly reduced (light-colored bars). In contrast, GO terms associated with proteostasis were enriched among HS-induced genes whose expression was enhanced by memory. To examine this further, we quantified the levels of Hsp12, a heat shock protein known to promote cell survival under various stress conditions, including elevated temperature, osmotic stress, and nutrient limitation. Unlike canonical molecular chaperones, Hsp12 primarily stabilizes the plasma membrane rather than assisting in protein folding. Hsp12 protein levels did not return to baseline after the recovery period. Instead, they remained at levels comparable to those at 20 min of heat shock, suggesting that Hsp12 degradation is delayed in memory conditions (Fig. 7b). These findings reveal a distinct persistence of Hsp12 protein during recovery, contrasting with the transient dynamics of trehalose accumulation.

### Mip6 functionally interacts with the histone deacetylase Rpd3

Rpd3 is a well-established regulator of transcriptional memory in *S. cerevisiae* (Lee *et al.* 2018). Mip6-TAP pulldown experiments conducted in our lab identified Rpd3 as a potential interacting partner (Supplementary File 4), suggesting a functional link between Mip6 and Rpd3. To explore this relationship, we first assessed whether Rpd3-regulated genes are overrepresented in specific expression response categories defined in our memory analysis (enhanced, dampened, or inverted; as in Figs. 5 and 6). Genes whose pattern is unaffected by memory were also included.

**Fig. 8.**
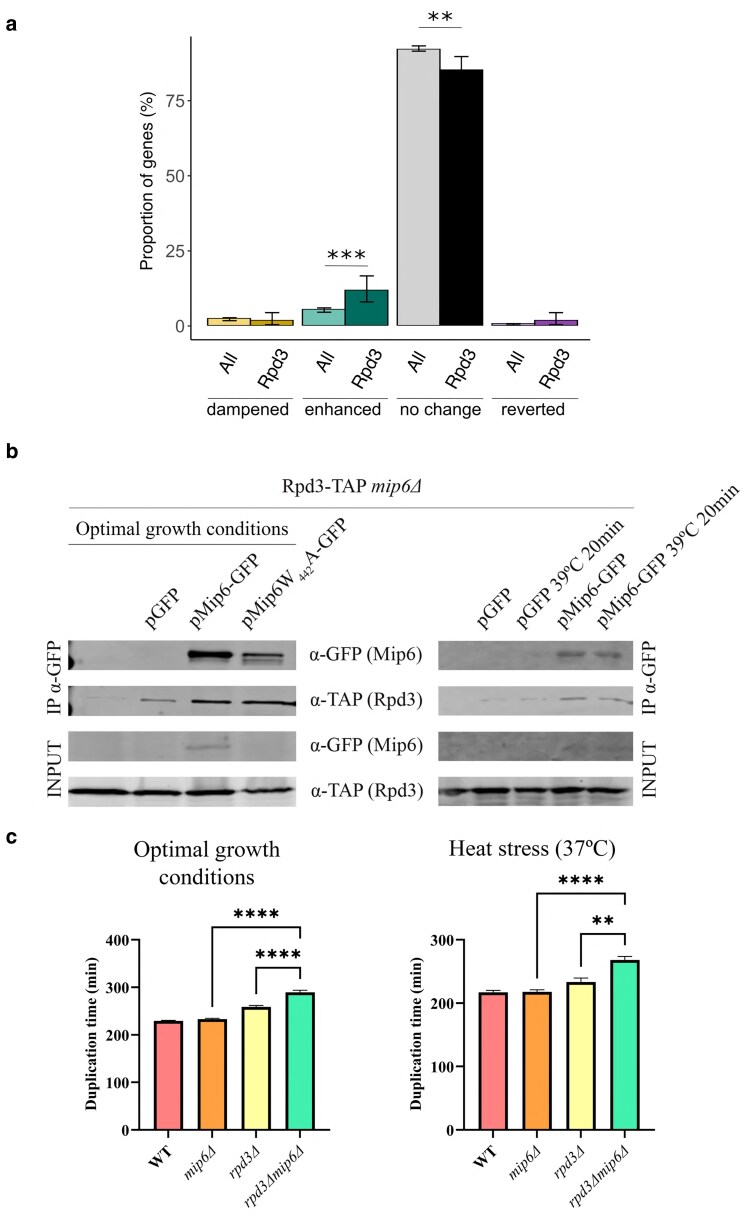
Mip6 physically, genetically, and functionally interacts with HDAC Rpd3. a) Comparison of the distribution of gene classification categories between the Rpd3-regulated gene set and all expressed genes. Bars represent the proportion of genes classified based on the effect of the memory × time × strain interaction factor as enhanced, dampened, no change, or inverted. Error bars indicate 95% confidence intervals, and statistical significance was assessed using a proportion test. Significance levels are indicated above bars: *P* < 0.001 (***), *P* < 0.01 (**), and *P* < 0.05 (*). A significant deviation from the overall gene distribution suggests enrichment or depletion of certain regulatory patterns within the Rpd3-regulated gene set. b) Immunoprecipitation of Mip6-GFP or Mip6W442A-GFP expressed from plasmid in Rpd3-TAP *mip6Δ* strain under optimal conditions or following a heat shock at 39°C for 20 min. An empty plasmid and a strain that expresses no plasmid were used as negative controls. Mip6-GFP and Rpd3-TAP were detected by Western blotting using the indicated antibodies. c) Duplication time of WT, *mip6Δ*, *rpd3Δ*, and *rpd3Δmip6Δ*, strains under optimal conditions and upon heat stress (37°C). Data represent the mean and the SE of 3 biological replicates. ****P-value < 0.001 (right).

Using the Saccharomyces Genome Database (SGD; https://www.yeastgenome.org) (Engel *et al.* 2025), we compiled a set of Rpd3-dependent genes and compared the distribution of their expression profiles to that of all expressed genes. As shown in Fig. 8a, the Rpd3-regulated gene set exhibited significant deviations from the overall distribution, suggesting selective enrichment or depletion of specific transcriptional memory patterns.

Given this enrichment and the prior identification of Rpd3 in the Mip6-TAP assay, we next tested whether Mip6 and Rpd3 physically interact. We performed a coimmunoprecipitation (co-IP) assay by expressing Mip6-GFP from a plasmid in an Rpd3-*TAP mip6Δ* strain under three conditions: optimal growth, heat shock (39°C for 20 min), and in the presence of the Mip6W442A point mutation, which disrupts the interaction between Mip6 and the mRNA export factor Mex67.

As shown in Fig. 8b, Mip6 copurifies with the histone deacetylase Rpd3 under all tested conditions, confirming a physical interaction between the 2 proteins. This interaction is preserved under both normal and heat stress conditions and occurs independently of Mex67 binding, as the Mip6W442A mutation does not impair Rpd3 copurification. These findings demonstrate that Mip6 interacts directly or indirectly with Rpd3 in a Mex67-independent manner, supporting a mechanistic link between Mip6 and chromatin-based regulation of transcriptional memory.

To further support a functional interaction between Mip6 and Rpd3, we analyzed the growth of WT, *mip6Δ*, *rpd3Δ*, and the double mutant *rpd3Δmip6Δ* strain under both optimal conditions and heat stress (37°C). Growth assays were conducted to compare growth curves and calculate doubling times across genotypes. As shown in Figs. 8b and 8c, the *rpd3Δmip6Δ* double mutant exhibited a significantly reduced growth rate compared to the corresponding single mutants, indicating a negative genetic interaction between *MIP6* and *RPD3*.

Both Mip6 and Rpd3 have previously been implicated in the regulation of Msn2/4-dependent transcripts (Ruiz-Roig *et al.* 2010; Martín-Expósito *et al.* 2019). To test whether the combined deletion of *MIP6* and *RPD3* exacerbates this regulatory effect, we measured the mRNA levels of a well-characterized Msn2/4 target gene, *HSP12*, in WT, *mip6Δ*, *rpd3Δ*, and *rpd3Δmip6Δ* strains using RT-qPCR.

**Fig. 9.**
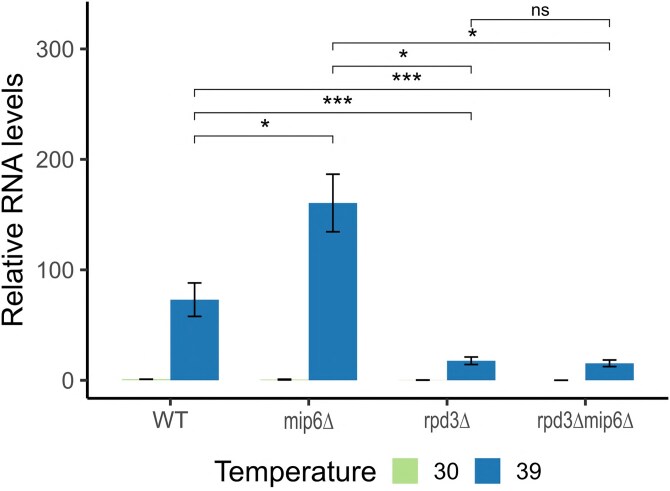
*HSP12* transcript levels in WT, *mip6Δ*, *rpd3Δ*, and *rpd3Δmip6Δ* strains under optimal and heat shock conditions. RNA was extracted from yeast cultures grown at 30°C or subjected to a 20-min heat shock at 39°C. *HSP12* expression was quantified by RT-qPCR and normalized within each biological replicate to the WT 30°C condition (set as 1). Bars represent the mean relative ###RNA levels across 3 biological replicates; error bars indicate standard error of the mean (SEM). Statistical significance was assessed using pairwise Wilcoxon tests comparing strains at 39°C, with significance levels denoted as *P* < 0.05 (), *P* < 0.01 (), *P* < 0.001 () and “ns” indicating nonsignificant differences.

As shown in Fig. 9, deletion of *MIP6* led to a significant increase in *HSP12* mRNA levels upon heat stress, consistent with a role for Mip6 in dampening stress-induced expression. In contrast, deletion of *RPD3* resulted in reduced expression of the transcript, and the *rpd3Δmip6Δ* double mutant exhibited mRNA levels comparable to the *rpd3Δ* single mutant. These results suggest that Rpd3 is epistatic to Mip6 and plays a dominant role in regulating Msn2/4-dependent gene expression during heat stress.

## Discussion

### Transcriptional memory dampens gene expression to prevent excessive activation or repression

Yeast cells display a transcriptional memory mechanism that enables a faster or altered response to heat stress following a prior exposure. This memory can persist across several generations (Light *et al.* 2013; D’Urso *et al.* 2016; Sump and Brickner 2022) and has also been documented in other organisms, including *Arabidopsis* (Ding *et al.* 2013; Liu *et al.* 2014). In yeast, most previous studies have focused on specific gene loci, such as those involved in galactose metabolism or *INO1* induction (Light *et al.* 2013; D’Urso *et al.* 2016; Randise-Hinchliff *et al.* 2016).

To examine the global transcriptional impact of heat shock memory, we designed an experiment in which cells were exposed to either a single heat shock (no-memory) or a sequential heat shock separated by a 60-min recovery at 30°C (memory condition). The temperature and recovery window were selected based on previous findings showing that stress-induced gene expression typically resets within 60 min (Lu *et al.* 2009), and because Mip6-containing stress granules also disassemble during this timeframe (Martín-Expósito *et al.* 2019). This setup allowed us to capture transcriptional and posttranscriptional changes associated with transcriptional memory and Mip6 function.

Our transcriptomic analyses led to several key conclusions. First, transcriptional memory predominantly dampens gene expression in response to heat stress, mitigating both excessive gene induction and repression upon reexposure. This buffering effect likely prevents overactivation of stress pathways, contributing to a more controlled adaptive response. The sustained accumulation of Hsp12 protein under memory conditions supports the idea that the sustained presence of stress-protective proteins reduces the need for strong transcriptional reinduction. Second, memory effects are gene-specific and enriched in biological processes such as metabolism, proteostasis, and reproduction, suggesting a selective role for memory in modulating essential adaptive pathways. Third, our data identify Mip6 as a key modulator of this response, acting in concert with the histone deacetylase Rpd3, a known chromatin-based regulator of transcriptional memory (Ruiz-Roig *et al.* 2010).

### Is Mip6 a negative regulator of HDAC Rpd3?

Mip6 is an RBP involved in regulating stress-responsive transcripts, including those associated with trehalose metabolism and ribosomal protein genes. While the molecular mechanism of Mip6's regulatory activity remains unclear, our results suggest that it may modulate chromatin structure indirectly via histone deacetylation. Specifically, Mip6 genetically interacts with the histone deacetylase Rpd3, suggesting a pathway centered on HDAC function.

We show that the *rpd3Δmip6Δ* double mutant exhibits synthetic growth defects under both optimal and stress conditions and that Mip6 physically interacts with Rpd3 regardless of temperature or Mex67-binding status. Moreover, Mip6 and Rpd3 affect overlapping transcriptional targets, including *HSP12* and *CTT1*, and the double mutant phenocopies the *rpd3Δ* strain, suggesting that Rpd3 is epistatic to Mip6. Together, these findings indicate that Mip6 may act as a negative regulator of Rpd3 activity or recruitment, potentially modulating histone deacetylation in a context-dependent manner. Interestingly, preliminary genetic data also suggest that Mip6 may positively regulate other HDACs, as shown by a synthetic genetic interaction with *hda1Δ*. This raises the possibility that Mip6 influences multiple deacetylase pathways, fine-tuning chromatin states across distinct stress-responsive gene sets depending on environmental context.

The precise nature of the Mip6–Rpd3 interaction remains unresolved. It is unclear whether Mip6 associates with all Rpd3-containing complexes or interacts specifically with a subcomplex. Notably, alternative forms of Rpd3, such as the stress-responsive isoform Rpd3μ, have been implicated in responses to osmotic and oxidative stress (Baker *et al.* 2013; McDaniel and Strahl 2013) and can be recruited to chromatin independently of Rpd3 core components. It will be important to determine whether Mip6 contributes to Rpd3 recruitment and interacts with other components of the Rpd3 regulatory complexes.

## Concluding remarks and broader implications

Together, our findings support a model in which transcriptional memory is governed by the interplay between RBPs and chromatin-based mechanisms. Mip6 appears to bridge posttranscriptional regulation and histone modification pathways, contributing to the fine-tuning of gene expression during repeated environmental challenges. Future studies should determine whether Mip6 interacts with specific Rpd3 complexes and whether it modulates histone deacetylation broadly or in a gene-specific manner. Expanding these analyses to other stress conditions may reveal a more general role for Mip6 in transcriptional memory and mRNA homeostasis. Understanding how yeast cells coordinate stress-induced gene expression and proteostasis in fluctuating environments could provide insights relevant to both microbial biotechnology and stress adaptation in higher eukaryotes, with potential biomedical applications.

## Supplementary Material

jkaf144_Supplementary_Data

## Data Availability

RNA-seq dataset is available at GEO as GSE276802. The R code for the analyses is available at https://github.com/SusanaRodriguezLab/TranscriptionalMemory/. Supplemental material available at *G3* online.
