# Supplementary material for: Transcriptional memory dampens heat shock responses in yeast: functional role of Mip6 and its interaction with Rpd3
Source: G3 (Bethesda). 2025 Jun 19;15(8):jkaf144. doi: 10.1093/g3journal/jkaf144 (PMC12341946; doi:10.1093/g3journal/jkaf144)
Supplement: jkaf144_Supplementary_Data [file jkaf144_supplementary_data.zip › Supplemental_Figure_S2_G3-2025-405979.pdf]

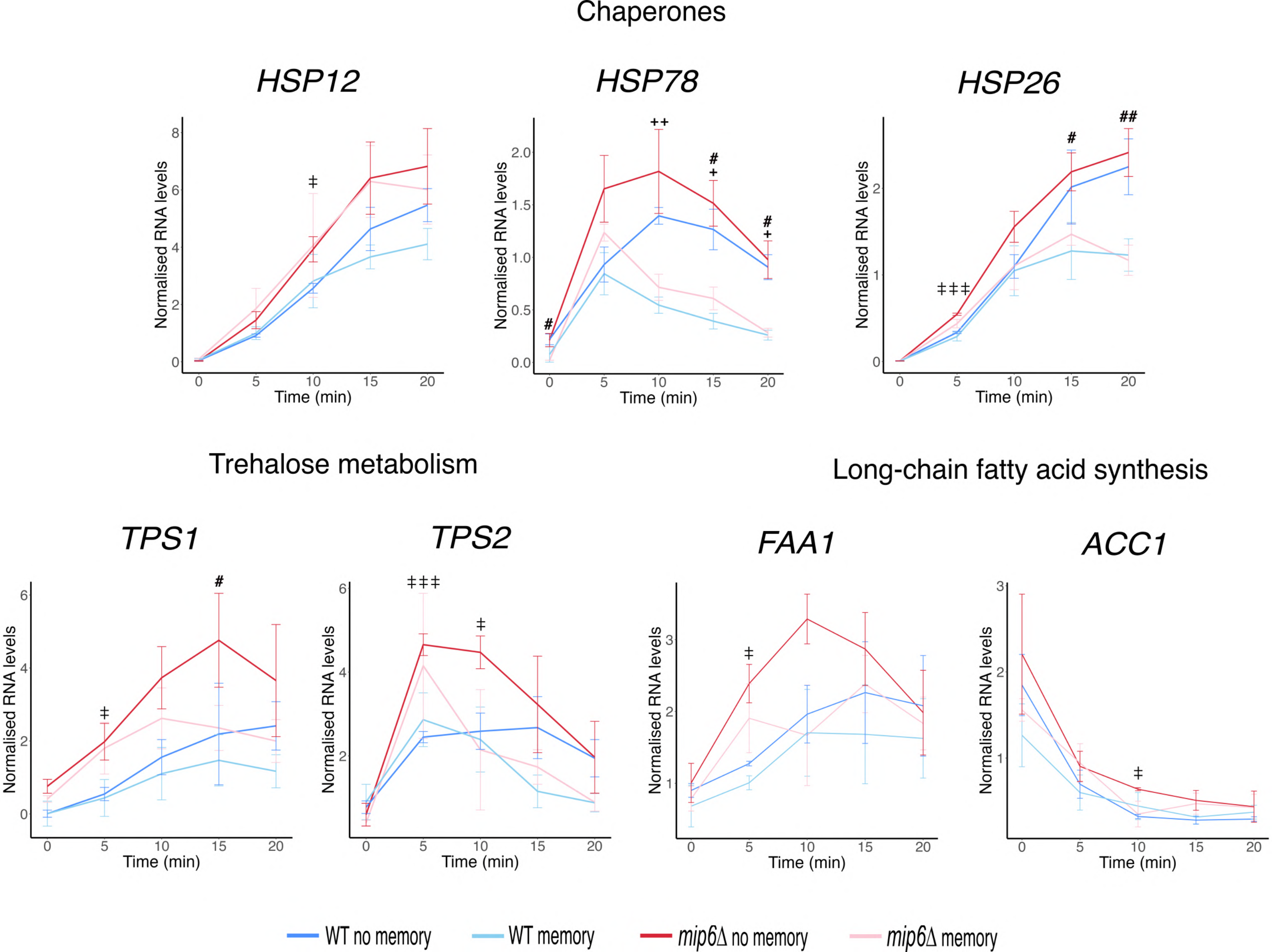
Chaperones
HSP12
HSP78
HSP26
Trehalose metabolism
Long-chain fatty acid synthesis
TPS1
TPS2
FAA1
ACC1
Normalised RNA levels
Time (min)
WT no memory
WT memory
mip6Δ no memory
mip6Δ memory
