# Supplementary material for: Transcriptional memory dampens heat shock responses in yeast: functional role of Mip6 and its interaction with Rpd3
Source: G3 (Bethesda). 2025 Jun 19;15(8):jkaf144. doi: 10.1093/g3journal/jkaf144 (PMC12341946; doi:10.1093/g3journal/jkaf144)
Supplement: jkaf144_Supplementary_Data [file jkaf144_supplementary_data.zip › Supplemental_File_S2_G3-2025-405979.pdf]

Dampened induction by memory

Strain-Attenuated Dampening of Induction Memory (n = 20)

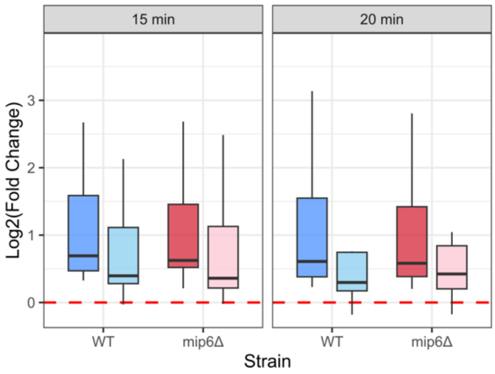

MGR1, HAC1, DUG1, CLF1, FBP1, LUC7, SND1, PKH3, DSF2, CRT10, YOR012W, VPS5, TRE2, GTT1, YPS6, ERR3, SAM50, YPL277C, ARO9, YEL073C

Strain-Exacerbated Dampening of Induction Memory (n = 40)

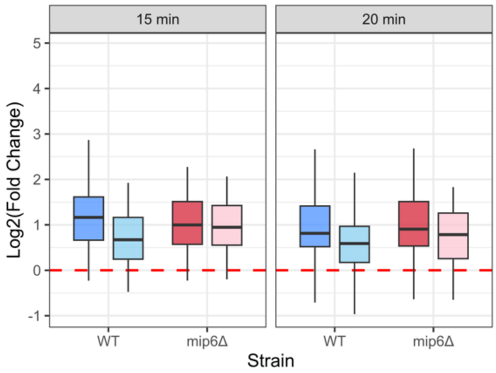

RRT12, IZH3, TML25, UIP3, STF1, CPP3, YNCD0013C, SAC6, YDR215C, PHO92, SNF1, GAL10, SHE10, YPS5, YGL258W-A, MCO32, YNCG0029C, SHY1, HUA1, MBB1, DAN4, ROD1, YNCI0010C, YIM1, YMR206W, GPI12, HER2, NPR1, YNCN0011W, YNL033W, PGC1, CAR1, YOP1, YHL042W, RPN3, ZRG8, UBP3, YER181C, CMC4, SNR6

Strain-Induced Inversion of Induction Memory (n = 3)

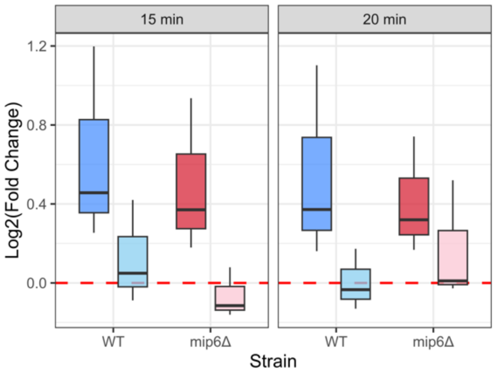

YNCF0008C, PTR3, IML1

Enhanced induction by memory

Strain-Attenuated Memory of Gene Induction (n = 7)

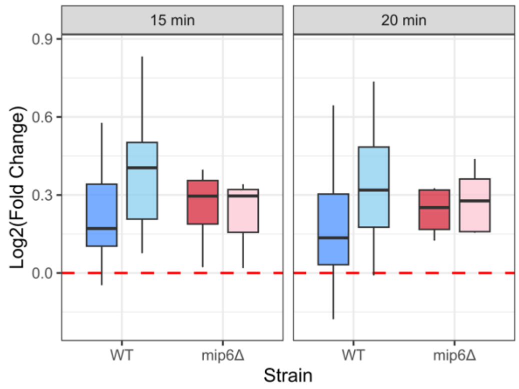

EPL1, JAC1, MRPS35, MRPL13, PPA2, COX11, SOL3

Strain-Enhanced Memory of Gene Induction (n = 3)

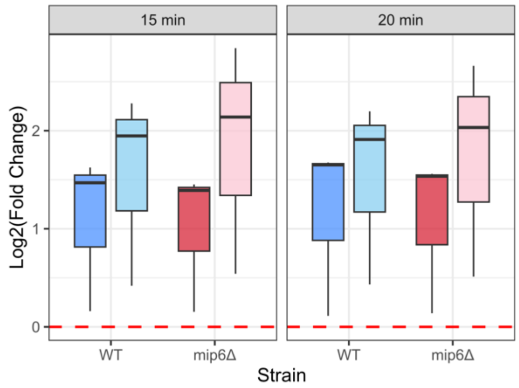

OTU1, ARC40, YOL038C-A

Strain-Induced Inversion of Induction Memory (n = 1)

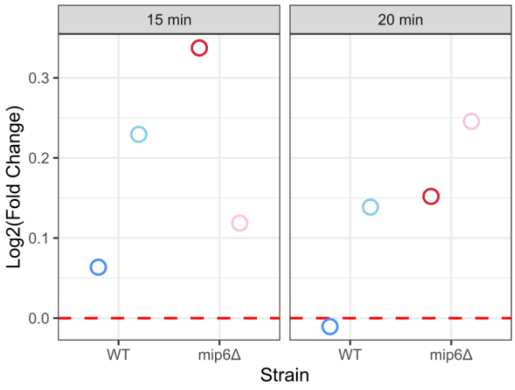

YPT6

Induction inverted by memory

Strain-Exacerbated Inversion of Induction Memory (n = 5)

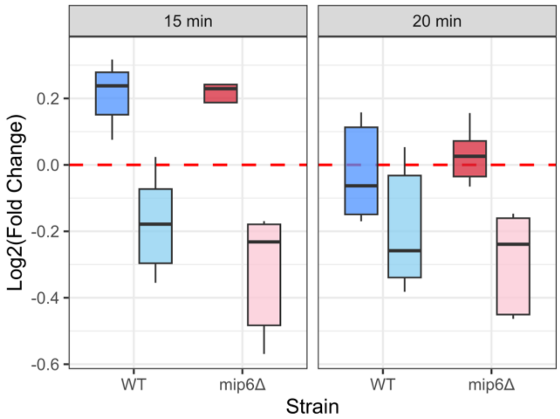

OCA4, TFC6, PET127, ORC1, RTT105

Strain-Attenuated Inversion of Induction Memory (n = 1)

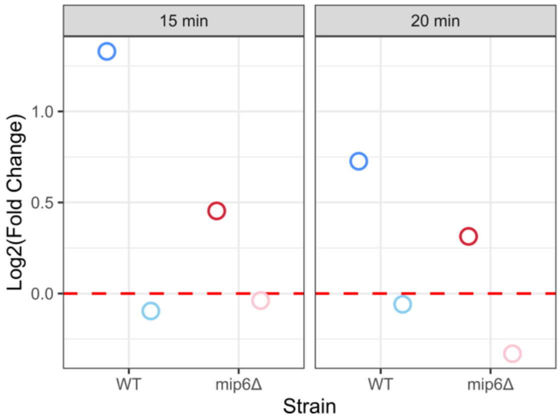

YNCP0008C

Enhanced repression by memory

Strain-Attenuated Memory of Gene Repression (n = 4)

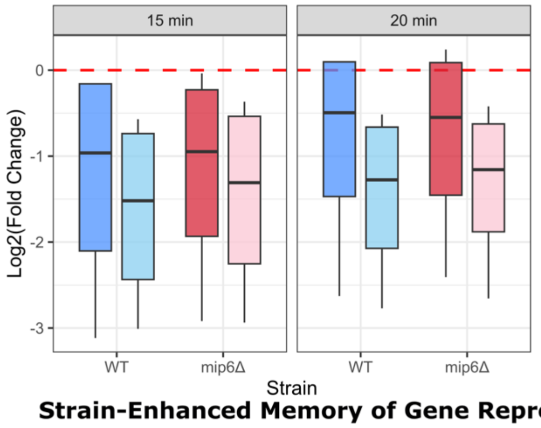

HAP4, YSR3, HRP1, SKS1

Strain-Enhanced Memory of Gene Repression (n = 5)

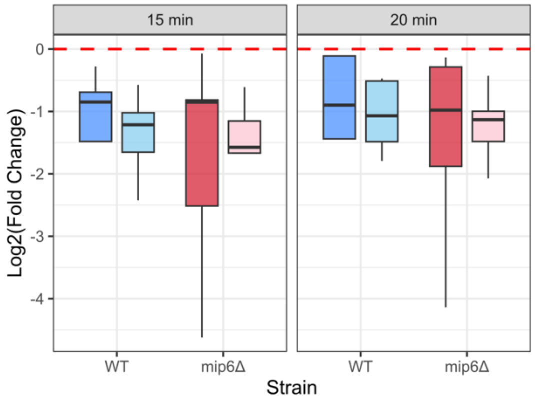

RPL15A, MNN14, YJR146W, CTF18, MUP3

Strain-Induced Inversion of Repression Memory (n = 1)

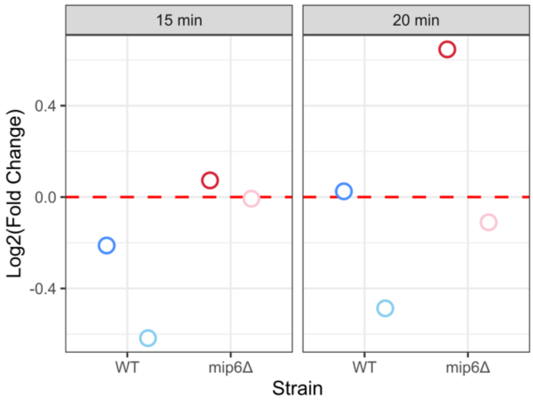

DIT2

Repression inverted by memory

Strain-Attenuated Inversion of Repression Memory (n = 3)

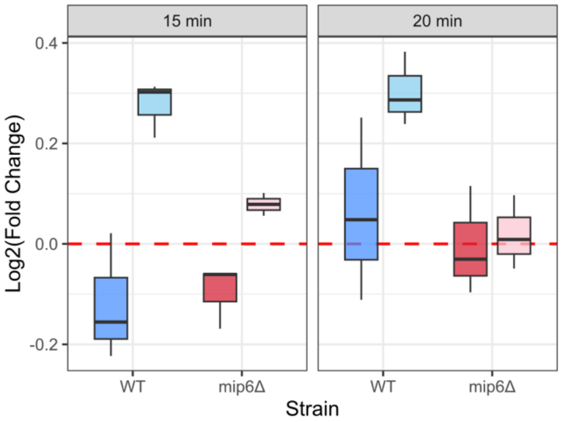

ROG1, MNP1, MRS3

Strain-Exacerbated Inversion of Repression Memory (n = 1)

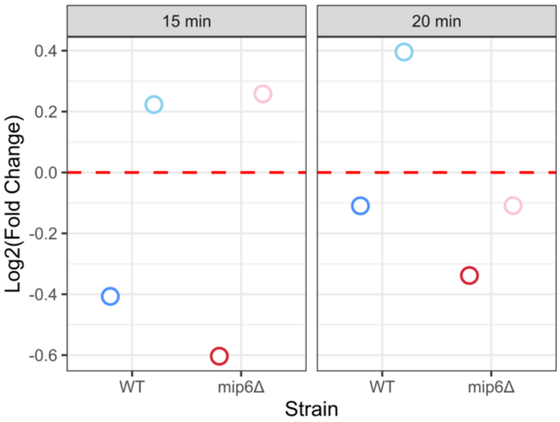

SNR31

Strain-Reverted Inversion of Repression Memory (n = 6)

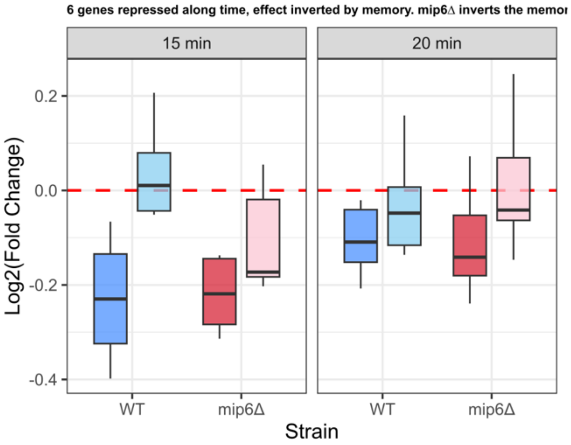

RSC20, JJ2, MSB4, TOP1, TAF7, ARP7

Dampened repression by memory

Strain-Exacerbated Dampening of Repression Memory (n = 4)

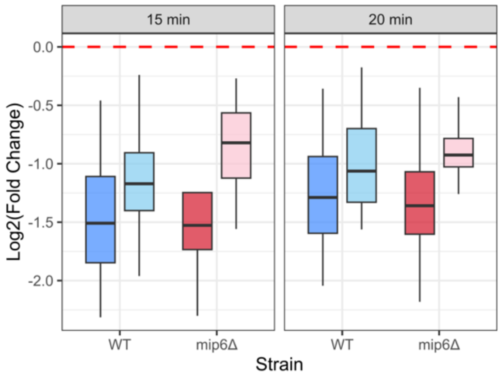

YBL029W, ORM1, URA2, GEP4

Strain-Attenuated Dampening of Repression Memory (n = 56)

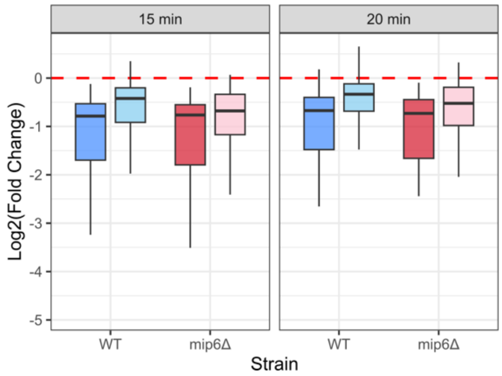

RPS14A, TAH1, YEH2, YLR179C, VTA1, MAP1, ADE1, CMR1, NSI1, ADK1, CIA1, HPT1, RPS18A, BNA4, ALG3, YBR197C, MTO1, TPN1, NUP57, SYF2, ADE3, SER2, SOP4, FAR1, ABF1, AUR1, PXL1, YAP7, HTZ1, ERP4, RTS2, SER1, RPL40A, CAB2, SER33, BET1, PRI1, ATR1, RPS18B, YML018C, PHA2, RPL18B, CBK1, RPL1A, SPT14, RPL33A, MNN9, AIM45, FHL1, RPL8A, RPS27B, TCA17, NPP2, IES5, SPT15, YER145C-A
