# Supplementary material for: Transcriptional memory dampens heat shock responses in yeast: functional role of Mip6 and its interaction with Rpd3
Source: G3 (Bethesda). 2025 Jun 19;15(8):jkaf144. doi: 10.1093/g3journal/jkaf144 (PMC12341946; doi:10.1093/g3journal/jkaf144)
Supplement: jkaf144_Supplementary_Data [file jkaf144_supplementary_data.zip › Supplemental_Material_G3-2025-405979.docx]

**Supplementary material legends**

**Figure S1.** Cell viability of memory and non-memory cultures subjected to heat shock in *WT* and *mip6Δ* strains. Viability was assessed by colony-forming units (CFU) on YPD plates incubated for 48 hours. Cells were sampled at 0 and 20 minutes of heat shock. The values shown represent CFU relative to the initial cell count. To facilitate comparisons, values were normalized to 1, representing the highest viability observed at each stress time point. Sample differences between memory and non-memory conditions, and between both strains (WT and *mip6∆*) were analyzed using a two-tailed paired and unpaired t-test in R, respectively. Data are presented as mean ± standard error (SE) from at least three biological replicates.

**Figure S2.** A 60-minute recovery prior to sequential heat exposure dampens the activation of heat shock genes. RNA levels of *HSP12*, *HSP78*, *HSP26*, *TPS*1, *TPS2*, *FAA1*, and *ACC1* were measured by qRT-PCR in WT and mip6Δ strains following the previously described experiment (Fig. 1). Gene expression values were calculated using the ΔΔCt method, with SCR1 as the reference gene. Data are presented as mean ± standard error (SE) from three biological replicates. Statistical comparisons were performed in R using two-tailed t-tests: (i) paired t-tests for WT memory vs. WT nomemory (p < 0.05 (+), p < 0.01 (++), p < 0.001 (+++)), (ii) paired t-tests for *mip6Δ* memory vs. *mip6Δ* no-memory (p < 0.05 (#), p < 0.01 (##), p < 0.001 (###)), and (iii) unpaired t-tests for WT vs. *mip6Δ* in the no-memory condition (p < 0.05 (‡), p < 0.01 (‡‡), p < 0.001 (‡‡‡)). The comparison between WT vs. *mip6Δ* in the memory condition showed no significant differences at any time point.

**File S1.** Categorization of genes according to model parameters

**File S2.** Expression and GO plots of genes belonging to each combination of parameter levels

**File S3.** Results from GO Enrichment analysis

**File S4.** Data from Mip6-TAP purification showing Rpd3S subunits.
