# Supplementary figures and images for: Transcriptional memory dampens heat shock responses in yeast: functional role of Mip6 and its interaction with Rpd3
Source: G3 (Bethesda). 2025 Jun 19;15(8):jkaf144. doi: 10.1093/g3journal/jkaf144 (PMC12341946; doi:10.1093/g3journal/jkaf144)

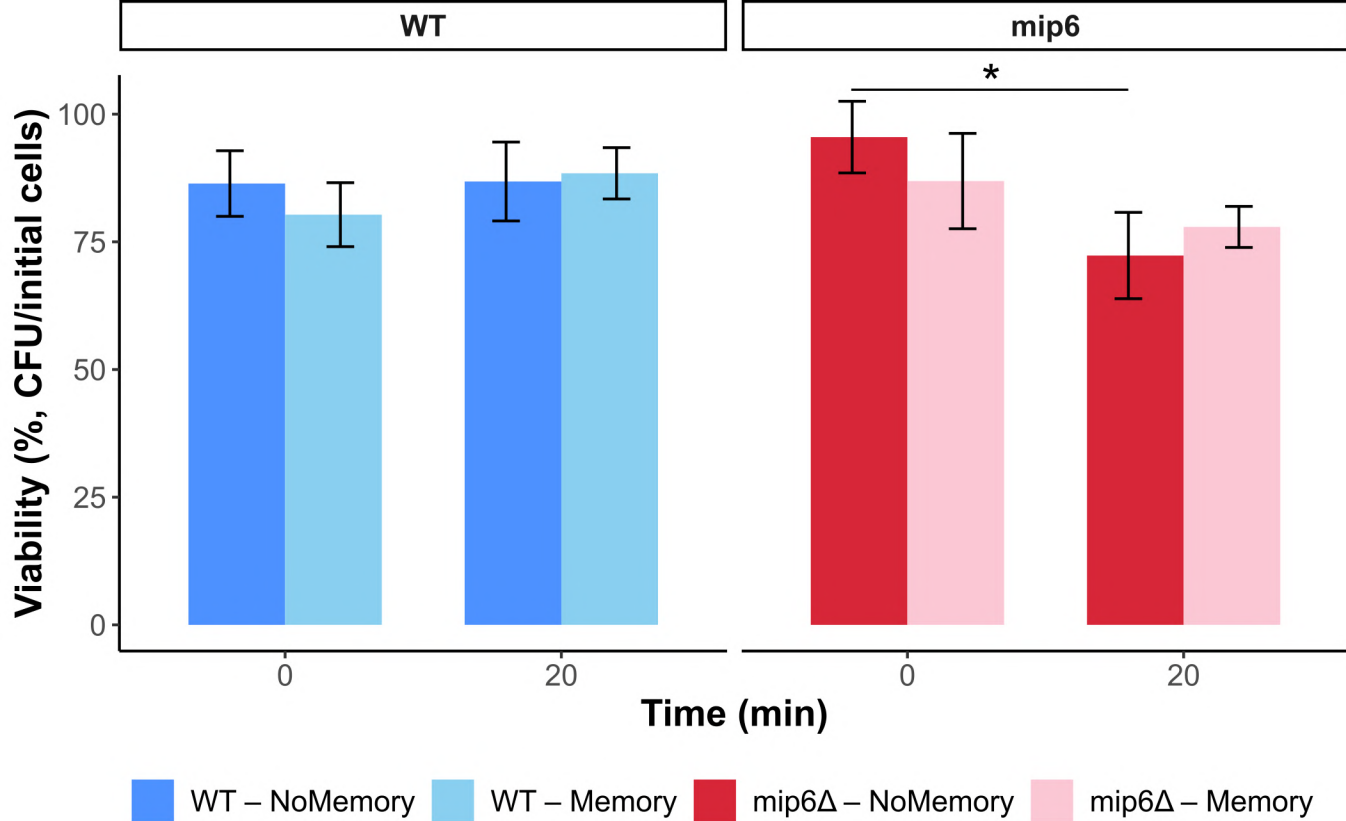

Supplement: jkaf144_Supplementary_Data [file jkaf144_supplementary_data.zip › Supplemental_Figure_S1_G3-2025-405979.pdf]
